# Avenanthramide C mitigates cisplatin-induced hippocampal neurotoxicity and cognitive impairment in rats via suppression of neuroinflammation and neuronal apoptosis

**DOI:** 10.3389/fphar.2025.1706224

**Published:** 2025-11-03

**Authors:** Maha Abdulrahman Aldubayan

**Affiliations:** Department of Pharmacology and Toxicology, College of Pharmacy, Qassim University, Buraidah, Saudi Arabia

**Keywords:** avenanthramide C, cisplatin, neuroinflammation, apoptosis, cognitive impairment, neurotoxicity

## Abstract

**Introduction:**

Cisplatin (CP)-induced cognitive impairment, commonly referred to as chemobrain, affects a substantial proportion of patients with cancer and currently lacks an effective pharmacological treatment. This condition is closely linked to neuroinflammation. Avenanthramide C (AVN-C), a bioactive compound uniquely found in oats, is known for its anti-inflammatory, anti-apoptotic, and neuroprotective properties. However, the precise mechanisms underlying its broader protective effects remain incompletely understood. This study aimed to investigate the potential of AVN-C to mitigate or prevent hippocampal damage in rats.

**Methods:**

Forty male Wistar rats were randomly divided into four groups (n = 10 per group): Control (5%DMSO/Saline), CP (8 mg/kg), AVN-C (6 mg/kg), and CP + AVN-C. AVN-C was administered orally once daily, while CP was delivered intraperitoneally on days 1, 4, and 7. Body weight and survival were monitored daily. Cognitive performance was assessed through behavioral tests, followed by biochemical analyses of hippocampal tissue. Inflammatory markers, NF-κB, TNF-α, IL-6, and IL-1β, and apoptotic markers (caspase-3 and BAX) were quantified.

**Results:**

CP administration resulted in significant reductions in body weight and survival. In contrast, co-treatment with AVN-C ameliorated these effects, markedly reducing hippocampal levels of NF-κB, TNF-α, IL-6, IL-1β, caspase-3, and BAX. Histopathologically, hippocampal tissues treated with CP + AVN-C were less damaged than tissues treated with the CP group. In conclusion, AVN-C significantly improved spatial learning and working memory in CP-treated rats and attenuated neuroinflammatory and apoptotic signaling.

**Discussion:**

These findings support the potential of AVN-C as a therapeutic agent for mitigating CP-induced neurotoxicity and cognitive dysfunction.

## Graphical Abstract

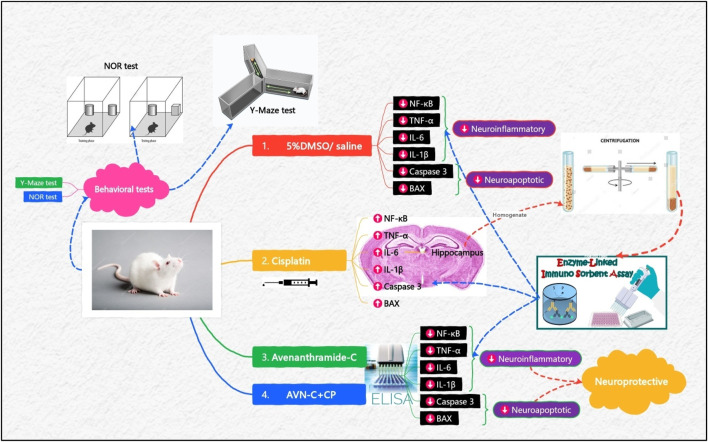

## 1 Introduction

Cognitive impairment is a notable adverse effect experienced by patients with cancer undergoing chemotherapy, affecting up to 75% of individuals during treatment and persisting for several months post-therapy (Alotayk et al., 2023; Alhowail, 2025; Janelsins et al., 2014). Several chemotherapeutic agents, including doxorubicin, methotrexate, cisplatin (CP), cyclophosphamide, 5-fluorouracil, and paclitaxel, have been implicated in chemotherapy-associated cognitive dysfunction (Alotayk et al., 2023; Aldubayan et al., 2024; Alsikhan et al., 2023). CP, a platinum-based anticancer agent, is widely used in the treatment of pediatric and adult cancers (Romani, 2022). Despite its clinical efficacy, its therapeutic utility is limited by severe adverse effects and toxicities affecting normal tissues (Qi et al., 2019). CP is particularly associated with learning and memory deficits, as demonstrated in both clinical and experimental studies (Mahmoud et al., 2023). Preclinical evidence indicates that CP increases peripheral inflammatory cytokines capable of crossing the blood–brain barrier, subsequently triggering the release of central pro-inflammatory mediators and amplifying neuroinflammatory responses (Jaiswara and Shukla, 2023; Wardill et al., 2016). Systemic inflammation induced by CP also compromises mitochondrial function (Alhowail, 2024), promoting the release of pro-apoptotic factors such as cytochrome c following mitochondrial DNA damage (Yang et al., 2014). This cascade activates caspases, ultimately leading to apoptotic cell death (Yang et al., 2014).

Although the mechanisms underlying CP-induced cognitive impairment remain incompletely understood, accumulating evidence suggests parallels with accelerated brain aging (Umfress et al., 2021). The hippocampus, a key region involved in attention, learning, and memory (Alhowail, 2025; Huo et al., 2018), is especially vulnerable to CP-induced toxicity. Dysregulation of hippocampal function has been linked to mild cognitive impairment and neurodegenerative diseases such as dementia (Park et al., 2024; Hanseeuw et al., 2016).

CP-induced neurotoxicity is associated with neuroinflammation (Alhowail, 2025). Neuroinflammation commonly arises in response to tissue injury or toxic insult (Khan and McLean, 2012). CP induces the expression of various pro-inflammatory cytokines and chemokines, including the nuclear translocation of the redox-sensitive transcription factor nuclear factor kappa B (NF-κB) (Ramesh and Reeves, 2002). Chronic inflammation plays a central role in the pathogenesis of neurodegenerative disorders through multiple converging pathways (Adamu et al., 2024). CP has been shown to elevate levels of tumor necrosis factor α (TNF-α) and interleukins (ILs) (Jang et al., 2021), particularly IL-6, both of which are critical mediators of CP-induced neurotoxicity (Hassan et al., 2024). Furthermore, TNF-α upregulates NF-κB activity, thereby amplifying neuroinflammation and promoting neuronal damage (Lawrence, 2009). CP accumulation in hippocampal cells also leads to mitochondrial dysfunction, increased lipid peroxidation, and excessive generation of reactive oxygen species (Abdel-Wahab and Moussa, 2019), contributing to redox imbalance, apoptotic signaling, and impaired cellular survival (Lomeli et al., 2017; Huang et al., 2024). CP further initiates the mitochondrial apoptotic cascade by modulating the expression of genes such as *p53*, *Bcl-2*, *Bax*, and various caspases (Li et al., 2024). Current clinical approaches to mitigate CP-induced neurotoxicity have shown limited efficacy (Alsaud et al., 2023), underscoring the need for effective neuroprotective interventions.

Avenanthramides (AVNs) are low molecular weight phenolic compounds derived from *Avena sativa* L. (oat grain) and are recognized for their potent antioxidant (Martínez-Villaluenga and Peñas, 2017; Aldubayan et al., 2019) and anti-inflammatory activities (Aldubayan et al., 2019; Wang and Eskiw, 2019; Peterson et al., 2002). Among these, avenanthramide-C (AVN-C) is the most abundant and exhibits the highest biological activity (Peterson et al., 2002; Perrelli et al., 2018; Xie et al., 2024). AVN-C suppresses the expression of pro-inflammatory cytokine genes in response to oxidative stress (e.g., H_2_O_2_) or TNF-α exposure (Wang and Eskiw, 2019), and has been shown to inhibit TNF-α signaling (Amir et al., 2019). Oat extracts enriched in AVNs reduce IL-6 and IL-8 release from endothelial cells stimulated with IL-1β (de Bruijn et al., 2019). Moreover, AVN-C attenuates oxidative stress, inflammation, and apoptosis in human skin fibroblasts (Wang and Eskiw, 2019; Pellegrini et al., 2016) and protects against CP-induced nephrotoxicity *in vivo* (Amir et al., 2019). Oral administration of AVN-C in a mouse model of Alzheimer’s disease improved cognitive performance and reduced neuroinflammation (Nathan et al., 2025). AVN-C exerts its anti-inflammatory effects through dual modulation of the NF-κB and Nrf2 signaling pathways. By inhibiting NF-κB activation and enhancing antioxidant defenses via Nrf2, AVN-C suppresses the expression of key pro-inflammatory cytokines (e.g., TNF-α, IL-6, IL-1β), thereby reducing neuroinflammation and neuronal apoptosis (Wang and Eskiw, 2019; Zhang et al., 2020). Notably, AVN-C crosses the blood–brain barrier, restores long-term potentiation, and reduces hippocampal neuroinflammation and apoptosis (Ramasamy et al., 2019; Lee et al., 2021; Chen et al., 2007).

Given the central role of the hippocampus in cognition and its high susceptibility to chemotherapeutic agents (Huo et al., 2018), no established treatment has effectively mitigated CP-induced cognitive deficits to date. Therefore, the present study aimed to evaluate the neuroprotective potential of AVN-C against cisplatin-induced cognitive dysfunction in rats. We conducted an assessment of behavioral changes utilizing the Y-maze and Novel Object Recognition (NOR) test. Additionally, we quantified hippocampal levels of key inflammatory markers, including NF-κB, IL-6, TNF-α, and IL-1β, as well as apoptotic markers such as BAX and caspase-3, through enzyme-linked immunosorbent assay (ELISA). Furthermore, histopathological staining of the hippocampus was performed to evaluate tissue structure and damage. In this proof-of-concept study, initial evidence is presented that highlights AVN-C as a promising therapeutic candidate for addressing chemotherapy-induced cognitive impairment, commonly referred to as “chemobrain,” by focusing on early molecular and behavioral changes. The findings may inform the development of novel, oat-derived neuroprotective strategies to combat chemotherapy-associated cognitive impairment. This study provides preliminary, yet novel, insight into the early effects of AVN-C in a rodent model of chemotherapy-induced cognitive impairment, offering a foundation for future long-term and mechanistic investigations.

## 2 Materials and methods

This study was designed to explore the early neuroprotective effects of AVN-C in a rat model of cisplatin-induced cognitive impairment. Behavioral assessments were conducted to detect cognitive and locomotor alterations, and hippocampal tissue was collected to evaluate neuroinflammatory and apoptotic responses at the molecular level. The tissues were subjected to histopathological staining.

### 2.1 Drugs

Cisplatin (1 mg/mL) was obtained from EBEWE Pharma Ges. m.b.H., Nfg. KG (Austria). AVN-C methyl ester (CAS No. 955382-52-2; Catalog No. CAY10011336-1) was procured from Cayman Chemical (Ann Arbor, MI, United States). AVN-C was dissolved in dimethyl sulfoxide (5% DMSO/Saline) before administration.

### 2.2 Animals

Forty male Wistar rats (200–250 g) were obtained from the College of Pharmacy, Qassim University. Upon arrival, animals were housed in standard polypropylene cages (4 rats per cage) with autoclaved wood chip bedding. Environmental conditions were maintained at a temperature of 25 °C ± 2 °C, relative humidity of 50–60%, and a 12-h light/dark cycle. Animals had free access to standard pellet chow and filtered tap water throughout the study. All animals were allowed to acclimatize to the housing conditions for 7 days before the start of experimental procedures. Survival rate was assessed daily, and body weight was recorded every other day to monitor general health status and treatment-related effects. All experimental procedures were reviewed and approved by the Animal Care and Use Committee of the Deanship for Scientific Research, Qassim University (Reference No. 23-67-07), and were conducted in strict accordance with institutional and national ethical guidelines for animal research.

### 2.3 Experimental design and drug administration

**SCHEME 1:**
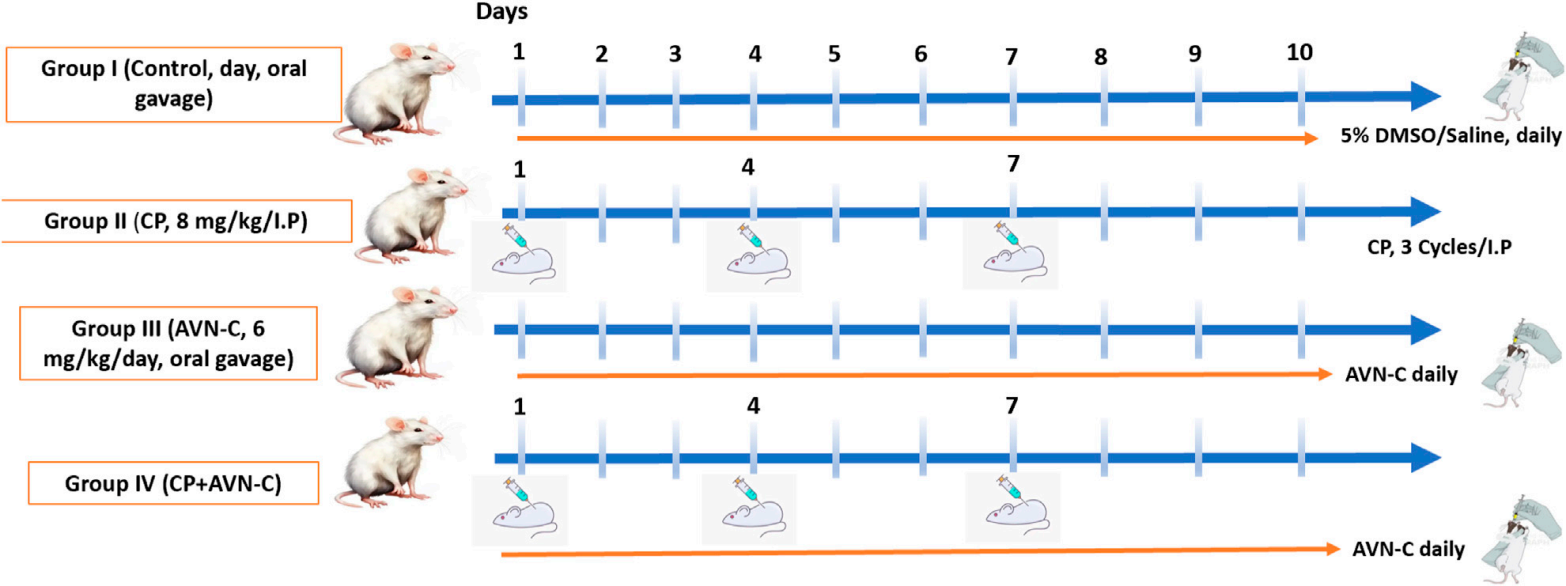
Schematic representation of the experimental design and results.

Animals were randomly divided into four groups (n = 10 per group). The control group received 5%DMSO/saline by oral gavage. The CP group received cisplatin (8 mg/kg, intraperitoneally) on days 1, 4, and 7 (Alhowail, 2025). The AVN-C group received AVN-C (6 mg/kg/day, orally via gavage) (Nathan et al., 2025). The CP + AVN-C group received cisplatin (8 mg/kg, intraperitoneally) on days 1, 4, and 7, in combination with AVN-C (6 mg/kg/day, orally) throughout the study. Cognitive function was assessed on days 10 and 11 using behavioral tests. Seven animals per group were evaluated, taking into account any mortality. Following behavioral assessments, hippocampal tissues were collected for enzyme-linked immunosorbent assay (ELISA)-based quantification of pro-inflammatory and apoptotic biomarkers (Scheme 1).

### 2.4 Survival rate and body weight

Survival was monitored daily, and deceased animals were promptly removed. Body weight was recorded every 3 days to track general health status and detect any adverse treatment-related effects.

### 2.5 Behavioral tests

#### 2.5.1 Y-maze

**SCHEME 2:**
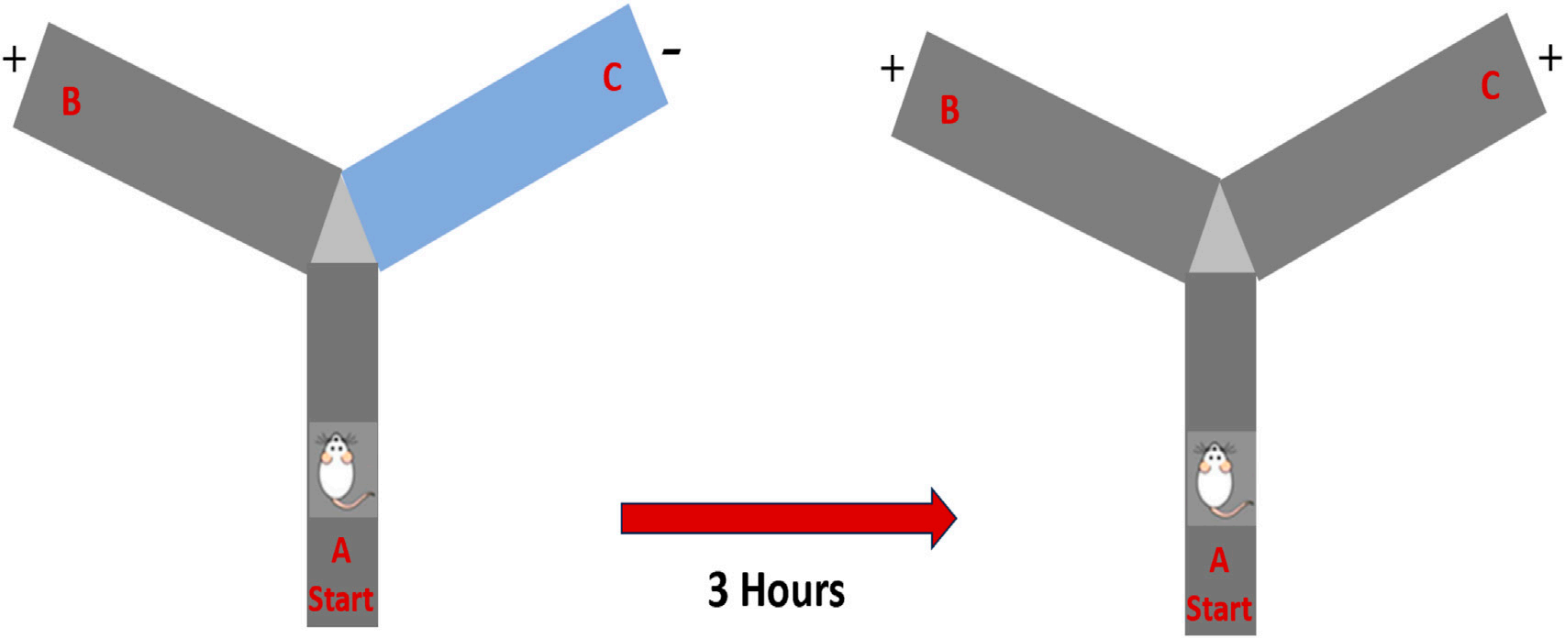
Schematic representation of the experimental design and drug administrations.

The Y-maze test was used to evaluate spatial learning and memory. The apparatus consisted of three wooden arms (50 cm × 10 cm × 18 cm), arranged at 120° angles. One arm was designated as the novel arm by occlusion during the training phase. Each rat was placed in the starting arm and allowed to explore the starting and familiar arms for 10 min. After a 3-h inter-trial interval, the test session was conducted, during which all three arms were accessible for 5 min. The rat was reintroduced to the start arm, and the time spent in the novel versus familiar arms, as well as the number of entries, was recorded. An entry was defined as the placement of all four paws within an arm (Alolayan and Alhowail, 2025) (Scheme 2).

#### 2.5.2 Novel object recognition (NOR)

**SCHEME 3:**
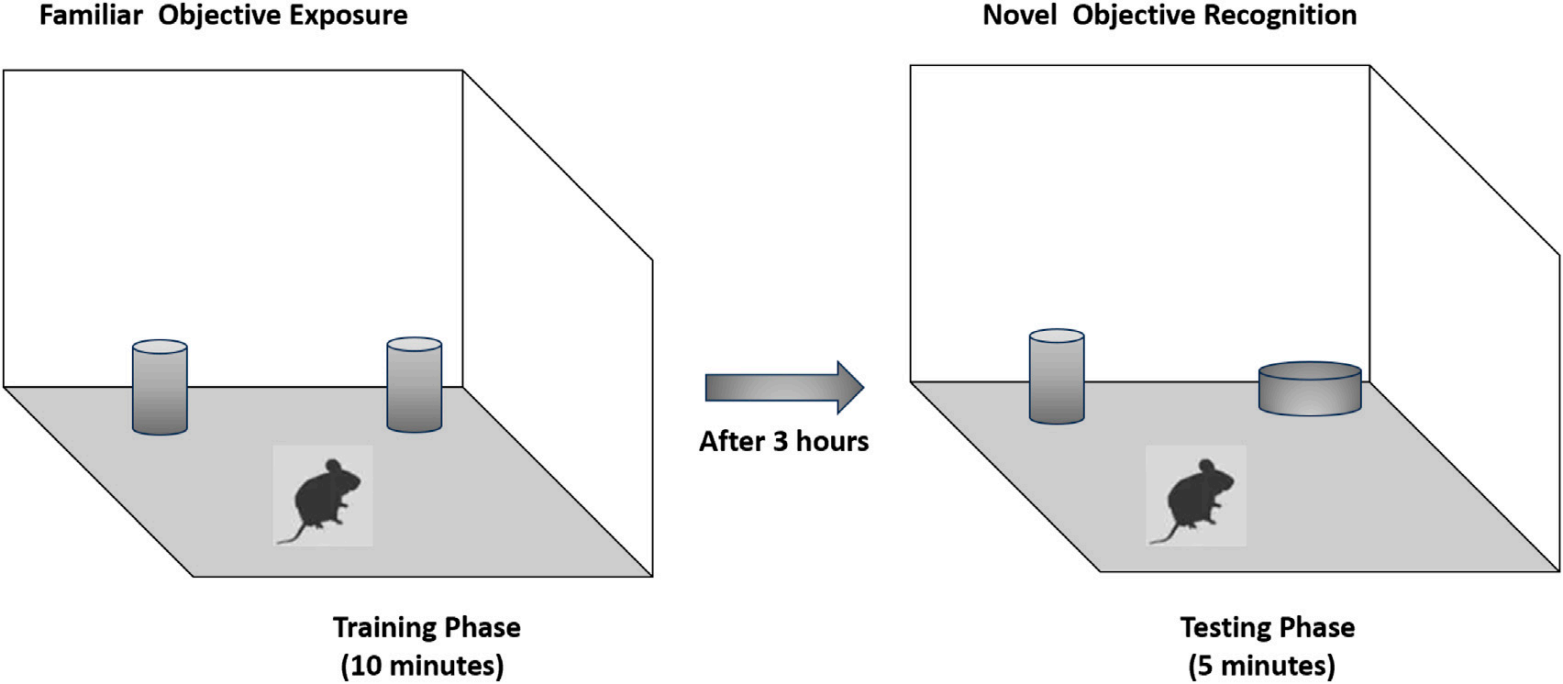
Schematic representation of behavior in the Y-maze.

Recognition memory was assessed using the novel object recognition test. The testing arena consisted of a wooden box (40 × 40 × 40 cm). During the training phase, two identical black cans were placed in opposite corners of the arena, equidistant from the walls to minimize location bias. Each rat was placed gently in the center of the box and allowed to explore the objects for 10 min. After a 3-h retention interval, one familiar object was replaced with a novel object—a white-painted reagent bottle—positioned in the same corner that the replaced object had previously occupied. The rat was then allowed to explore for 5 min. The total interaction time with the novel and familiar objects was recorded and analyzed via video tracking software by an observer blinded to the experimental groups. To prevent olfactory cues from influencing behavior, the arena and objects were cleaned thoroughly with 70% ethanol and allowed to dry between trials (Alhowail and Aldubayan, 2023) (Scheme 3).

### 2.6 Hippocampal tissue collection for biochemical analysis

Rats were euthanized with CO_2_ in a glass chamber (Alhowail, 2025). Immediately after decapitation, the skull was carefully opened using surgical scissors, and the whole brain was rapidly removed and placed on an ice-cold glass plate. Under proper lighting and with the aid of anatomical landmarks, the hippocampus was carefully dissected from each hemisphere by gently removing the overlying cortical tissue. The isolated hippocampi were then homogenized using a Qsonica homogenizer (30 Hz, Newtown, CT, United States) in conjunction with N-PER lysis buffer (Thermo Scientific, Madison, WI, United States) and centrifuged at 12,000 rpm for 10 min. The resulting supernatants were collected and stored at −80 °C for subsequent analysis.

### 2.7 Enzyme-linked immunosorbent assay

Inflammatory and apoptotic markers in the hippocampal tissue supernatants were quantified using ELISA. The following rat-specific ELISA kits (ABclonal Technology, Woburn, MA, United States) were used: IL-1β (Cat. No. RK00009), IL-6 (Cat. No. RK00020), NF-κB (Cat. No. RK08775), TNF-α (Cat. No. RK00029), BAX (Cat. No. RK03522), and caspase-3 (Cat. No. RK03549). Absorbance was measured at 450 nm using a microplate reader (BioTek Instruments, United States) (Rana and Soni, 2008).

### 2.8 Histopathological evaluation of hippocampal tissue

Brains were rapidly removed and placed on an ice-cold dissection plate. The hippocampal region was carefully excised under a stereomicroscope, guided by anatomical landmarks. Tissue samples were immediately fixed in 10% neutral-buffered formalin for 24–48 h and embedded in paraffin wax. Coronal sections (5 µm) were cut using a rotary microtome and mounted on glass slides. Sections were stained with hematoxylin and eosin (H&E) for light microscopic examination. Histopathological changes, including neuronal degeneration, vacuolation, and nuclear pyknosis, were evaluated under ×40 magnification.

### 2.9 Statistical analysis

Data were analyzed using GraphPad Prism 9 (GraphPad Software, La Jolla, CA, United States). One-way analysis of variance (ANOVA) followed by Tukey–Kramer *post hoc* testing was performed for multiple comparisons. A *p*-value <0.05 was considered statistically significant. Data are presented as mean ± standard error of the mean (SEM).

## 3 Results

### 3.1 Effect of CP on survival

**FIGURE 1:**
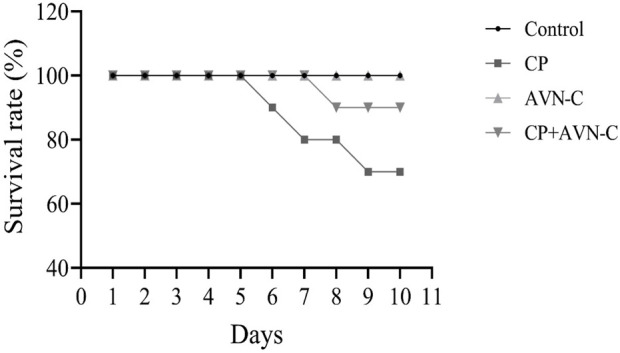
Survival rate of rats following cisplatin (CP) treatment, with or without avenanthramide C (AVN-C) co-administration.

Cisplatin treatment resulted in 30% mortality by day 10, whereas the co-administration of AVN-C reduced mortality to 10%, compared to 0% in the control group (Figure 1).

### 3.2 Effect of CP on body weight

**FIGURE 2:**
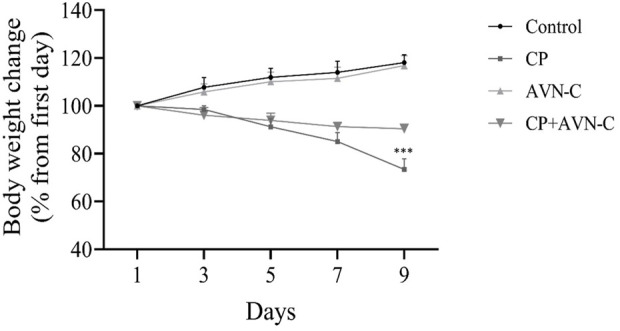
Effect of CP and AVN-C on body weight. Statistical analysis was performed using one-way ANOVA with Tukey–Kramer *post hoc* test. CP ***p < 0.001 vs. control or CP + AVN-C group.

Body weight was recorded on days 0, 3, 6, and 9. CP-treated rats exhibited a significant reduction in body weight on days 6 and 9 compared to both the control and CP + AVN-C groups (Figure 2).

### 3.3 Effect of CP and AVN-C on Y-maze performance

**FIGURE 3:**
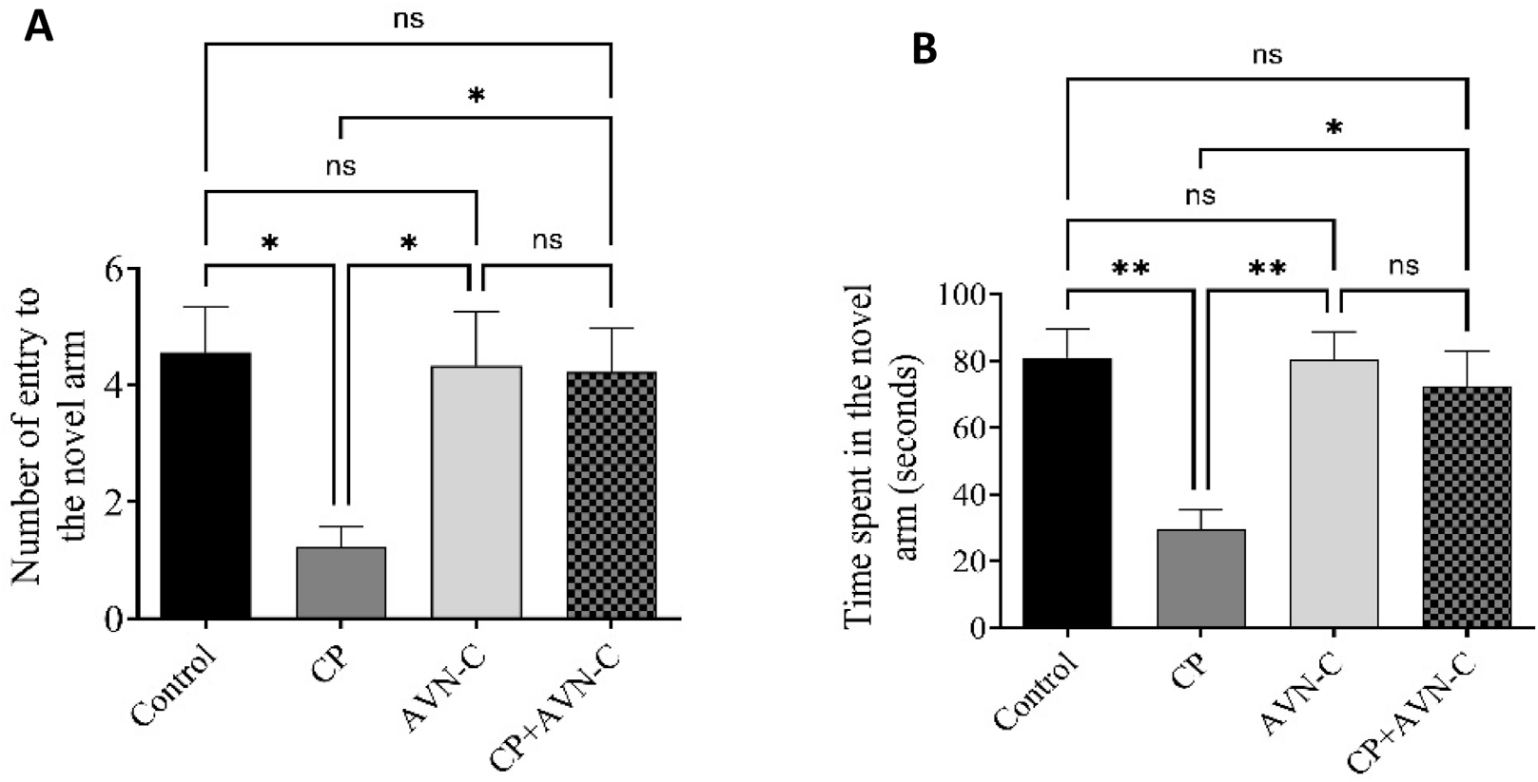
Effect of CP and AVN-C on spatial working memory, assessed by the Y-maze test. **(A)** Number of entries into the novel arm. **(B)** Time spent in the novel arm. Data are presented as mean ± standard error of the mean (SEM) (n = 7). Statistical analysis was performed using one-way analysis of variance (ANOVA) followed by Tukey–Kramer *post hoc* test. *p < 0.05, **p < 0.01 vs. control or CP groups.

In the Y-maze test, control rats demonstrated a higher number of entries into the novel arm compared to all other groups. CP-treated rats exhibited the lowest number of novel arm entries (Figure 3A), indicating impaired spatial working memory. AVN-C treatment significantly increased both the number of entries and the time spent in the novel arm relative to CP alone (Figure 3B), suggesting improved cognitive performance.

### 3.4 Effect of CP and AVN-C on the NOR test

**FIGURE 4:**
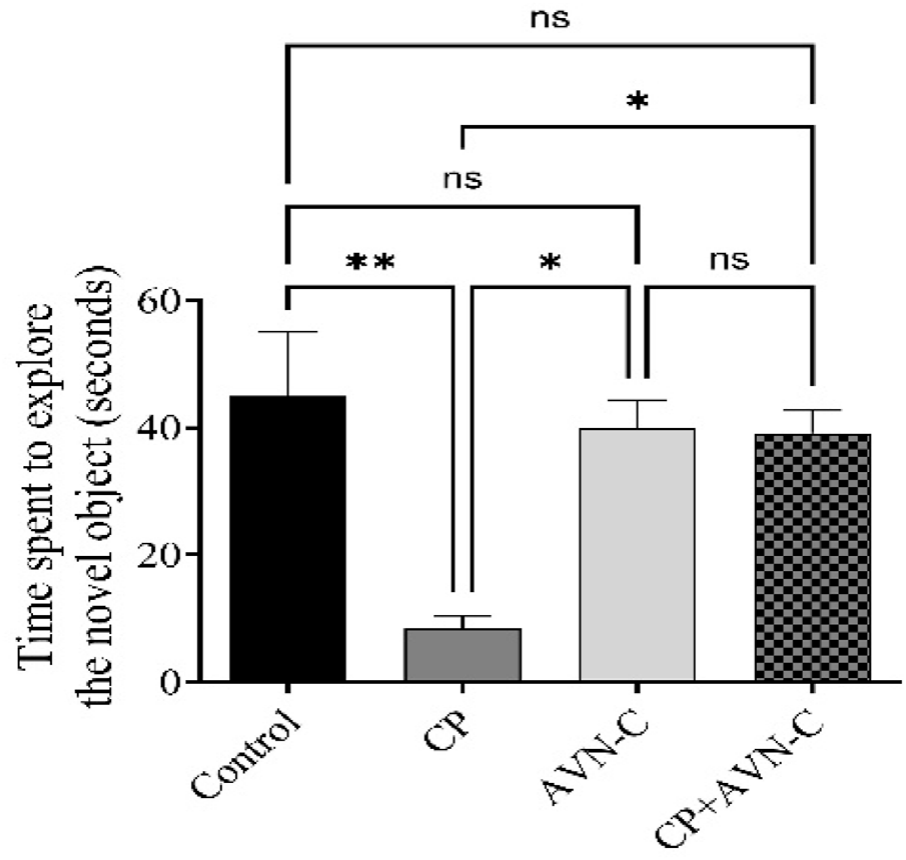
Effect of AVN-C on CP-induced cognitive deficits in the novel object recognition (NOR) test. AVN-C mitigated CP-induced reductions in exploration time of the novel object. Data are presented as mean ± SEM (n = 7). Statistical significance was determined using one-way ANOVA followed by Tukey–Kramer *post hoc* test. *p < 0.05, **p < 0.01 vs. control or CP groups.

CP-treated rats spent significantly less time exploring the novel object compared to controls (Figure 4), indicating memory impairment, and co-treatment with AVN-C significantly increased novel object exploration time, reflecting improved recognition memory and a reversal of CP-induced deficits.

### 3.5 Effect of CP and AVN-C on inflammatory markers in the hippocampus

**FIGURE 5:**
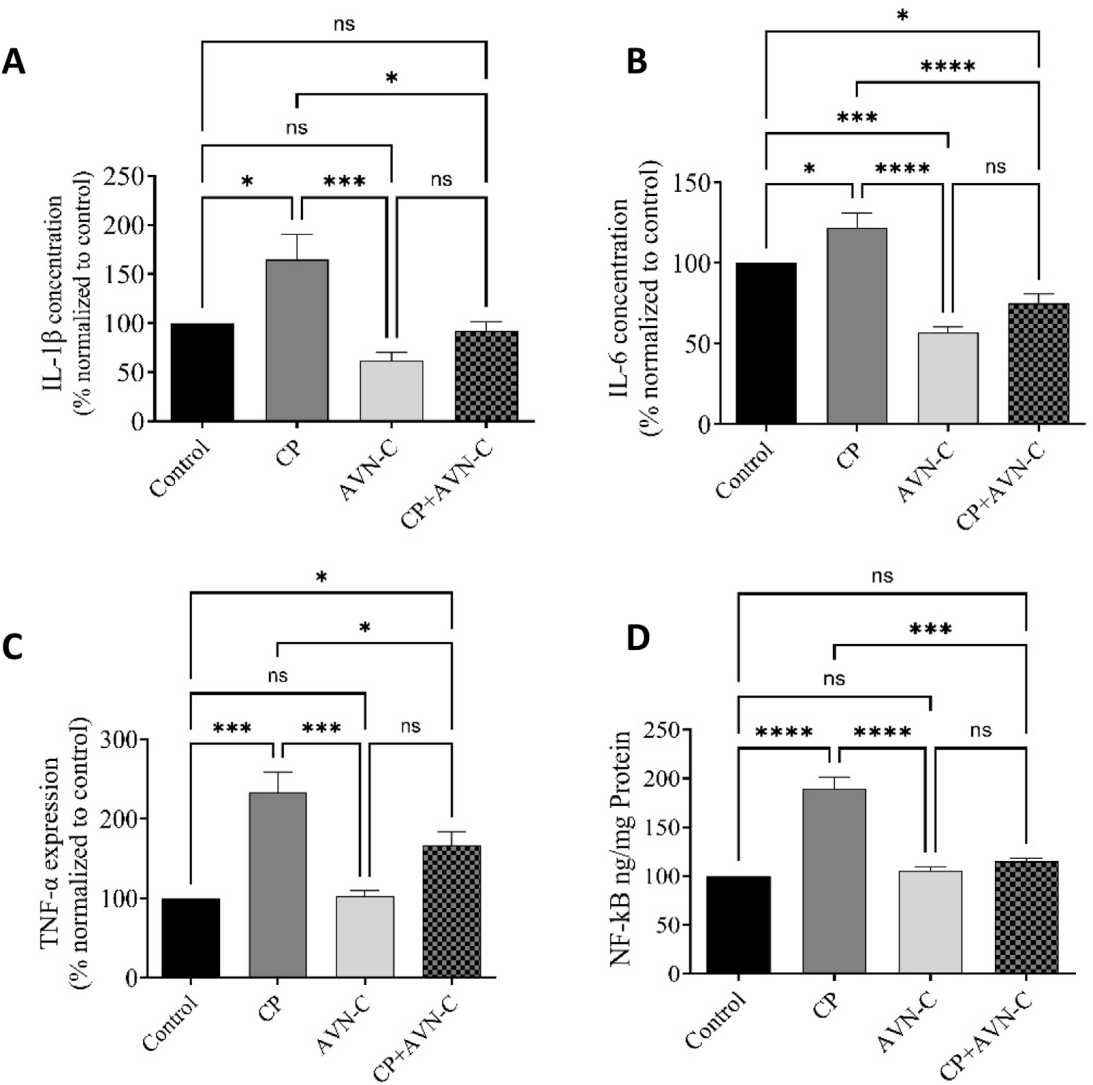
Effect of AVN-C on CP-induced changes in pro-inflammatory cytokine levels in rat hippocampal tissue. **(A)** IL-1β, **(B)** IL-6, **(C)** TNF-α, **(D)** NF-κB. Data are presented as mean ± SEM (n = 7). Statistical analysis was performed using one-way ANOVA with Tukey–Kramer *post hoc* test. *p < 0.05, ***p < 0.001, ****p < 0.0001 vs. control or CP group.

CP-treated rats presented significantly elevated hippocampal levels of IL-1β, IL-6, TNF-α, and NF-κB compared to controls (Figures 5A–D). Co-administration with AVN-C markedly reduced the expression of all four inflammatory markers, indicating an anti-inflammatory effect in the hippocampus.

### 3.6 Effect of CP and AVN-C on apoptotic markers in the hippocampus

**FIGURE 6:**
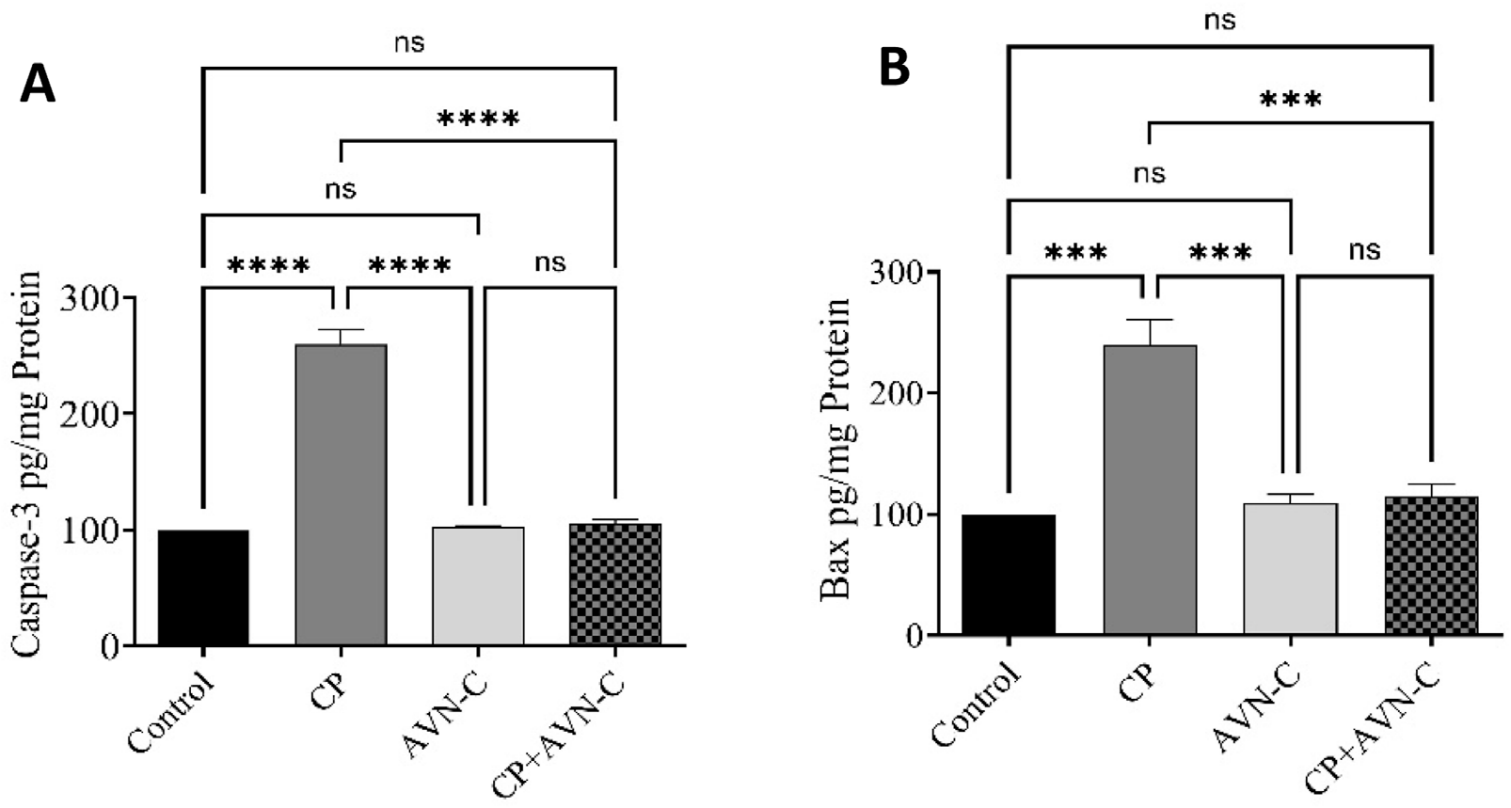
Effect of AVN-C on CP-induced alterations in hippocampal apoptotic markers. **(A)** Caspase-3, **(B)** BAX. Data are presented as mean ± SEM (n = 7). Statistical analysis was performed using one-way ANOVA followed by Tukey–Kramer *post hoc* test. ***p < 0.001, ****p < 0.0001 vs. control or CP group.

Hippocampal levels of caspase-3 and BAX were significantly elevated in CP-treated rats relative to controls (Figures 6A,B). AVN-C co-treatment significantly reduced the expression of both apoptotic markers, suggesting an anti-apoptotic effect.

### 3.7 Histological staining

**FIGURE 7:**
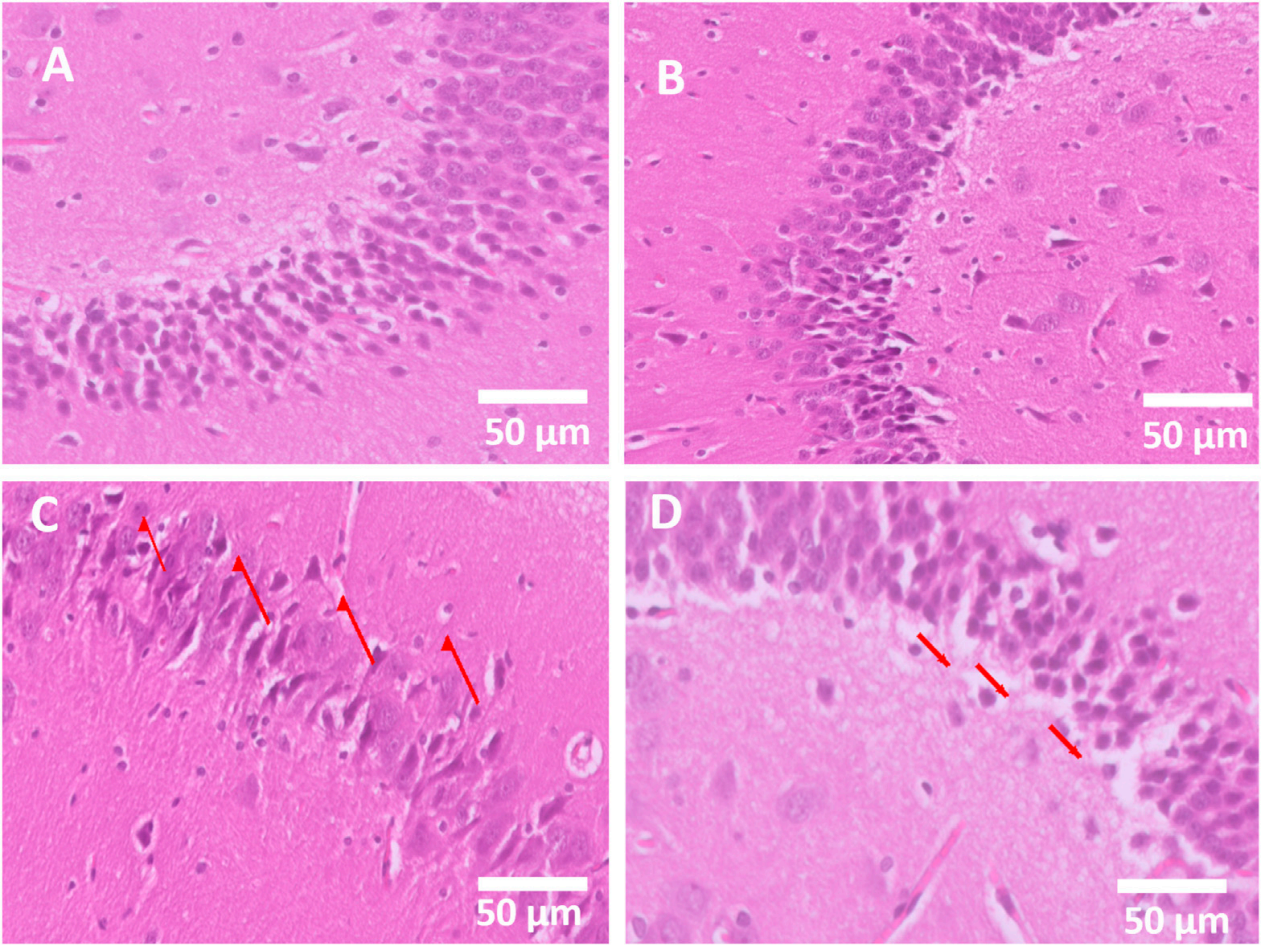
Histopathological evaluation of hippocampal neurons (H&E, 50 µm). **(A)** The control group shows standard neuronal architecture (score 0, normal). **(B)** The AVN-C group displays a preserved neuronal structure similar to that of the control (score 0, normal). **(C)** The cisplatin group exhibits severe neuronal degeneration and vacuolation (score 3, severe damage). **(D)** Cisplatin + AVN-C group demonstrates partial neuronal protection with reduced degeneration (score 1–2, mild to moderate damage).

Hippocampal neuron sections were examined by light microscopy, and it was observed that the control exhibited a normal hippocampal neuronal architecture with intact cell density. The AVN-C-treated group showed a neuronal structure preserved, comparable to the control (Figures 7A,B). However, the CP group exhibited marked neuronal degeneration, cell shrinkage, vacuolation, and pyknotic nuclei (arrows) (Figure 7C). In contrast, the CP + AVN-C treatment exhibited partial preservation of neurons, reduced degeneration compared to cisplatin alone (arrows indicate mild neuronal loss) (Figure 7D).

## 4 Discussion

This study was conducted to assess whether AVN-C, a natural antioxidant derived from oats, can mitigate early cognitive and molecular changes induced by cisplatin treatment in rats. Our findings demonstrate that AVN-C alleviated behavioral deficits and significantly reduced hippocampal inflammation and apoptosis markers, supporting its potential as a neuroprotective candidate in the context of chemotherapy (CP)-induced cognitive dysfunction. To establish a chemobrain model, CP was administered and its effects evaluated through behavioral tests. In the Y-maze test, CP treatment impaired spatial memory, as evidenced by a reduced number of entries into novel arms. In contrast, co-administration of AVN-C increased both the number of entries and the time spent in the novel arm, indicating preserved spatial memory (Liet et al., 2015).

Similarly, in NOR test, CP-treated rats exhibited diminished discrimination between familiar and novel objects, consistent with earlier reports of CP-induced deficits in working memory (Alhowail, 2025). Co-treatment with AVN-C significantly increased exploration time of the novel object, suggesting that AVN-C counteracted CP-induced recognition memory impairments. The protective effect of AVN-C may be attributed to early and sustained administration, which limits microglial overactivation and preserves microglial phagocytic function. This effect aligns with previous findings demonstrating that AVN-C improves recognition memory and prevents synaptic plasticity impairment in amyloid β-treated animals (Nathan et al., 2025; Ramasamy et al., 2019).

In addition to cognitive outcomes, CP administration resulted in a significant reduction in final body weight compared to the control group. This finding is consistent with previous studies showing persistent growth impairment in CP-treated rats despite resumption of weight gain after treatment cessation (Mokhtar et al., 2021). CP-induced weight loss is commonly attributed to its emetogenic properties, which lead to reduced appetite, gastrointestinal toxicity, and diarrhea (He et al., 2023). AVN-C administration alleviated weight loss, likely due to its anti-inflammatory and anti-apoptotic actions.

Mechanistically, AVN-C exerted its protective effects by modulating neuroinflammatory and apoptotic pathways. CP administration significantly upregulated NF-κB and increased hippocampal levels of TNF-α, IL-1β, and IL-6, indicating robust neuroinflammation. These results are consistent with evidence implicating NF-κB-dependent signaling in the pathogenesis of chemobrain (Bagnall-Moreau et al., 2019). AVN-C co-treatment significantly reduced NF-κB expression and suppressed the associated proinflammatory cytokines, thereby attenuating neuroinflammation. These findings align with reports that AVN-C preserves cognitive function by inhibiting NF-κB-mediated cytokine release, a mechanism also relevant to Alzheimer’s disease pathology (Nathan et al., 2025).

Apoptosis also plays a key role in CP-induced neurotoxicity (Lomeli et al., 2017). In this study, AVN-C reduced hippocampal expression of the apoptotic markers caspase-3 and BAX, indicating protection against CP-induced neuronal apoptosis.

In addition, the histopathological evaluation of the hippocampus further supports the neuroprotective effect of AVN-C against CP-induced neuronal injury. In the control and AVN-C alone groups, neurons exhibited preserved architecture with no evidence of degeneration (score 0). In contrast, the CP group demonstrated severe neuronal damage, characterized by loss of normal cytoarchitecture, vacuolation, and neuronal shrinkage (score 3), consistent with previous reports of CP-induced neurotoxicity (Kandeil et al., 2020). Importantly, co-treatment with AVN-C significantly attenuated these pathological alterations, as reflected by reduced neuronal degeneration and preservation of hippocampal organization (score 1–2). These findings suggest that AVN-C confers structural neuroprotection, likely by mitigating inflammation and apoptotic signaling pathways triggered by CP. The partial rescue of hippocampal neurons by AVN-C aligns with the behavioral and biochemical data, highlighting its therapeutic potential in preserving cognitive function during CP chemotherapy.

This study has several strengths, including the consistent use of rat strain, age, and sex, as well as the novelty of assessing both inflammatory and apoptotic markers in the hippocampus following AVN-C + CP treatment. To date, no previous studies have specifically investigated the neuroprotective effects of AVN-C against CP-induced toxicity. A limitation of the study, despite the novel findings presented, this study has several limitations. First, the experimental timeframe was relatively short, with behavioral and molecular assessments conducted within 10–11 days post-treatment with CP. This limits our ability to evaluate the long-term persistence of cognitive deficits and the sustained neuroprotective effects of AVN-C. Second, although we assessed key inflammatory and apoptotic markers at the molecular level, protein-level validation (e.g., via Western blot or immunohistochemistry) was not performed. Additionally, a limitation is the omission of spontaneous alternation percentage in the Y-maze, which is a more sensitive indicator of working memory. Finally, while AVN-C showed promising effects, its mechanism of action remains incompletely understood and requires further investigation using more targeted molecular and cellular assays.

In summary, this study demonstrates that CP induces cognitive deficits associated with increased hippocampal levels of inflammatory markers (IL-1β, IL-6, NF-κB, and TNF-α) and apoptotic markers (caspase-3 and BAX). AVN-C supplementation significantly mitigated these effects, as evidenced by improved performance in behavioral tests, enhanced spatial learning and working memory, and reductions in neuroinflammatory and apoptotic signaling. Histopathologically, hippocampal tissues treated with CP + AVN-C were less damaged than tissues treated with the CP group. Collectively, these findings highlight the therapeutic potential of AVN-C as a protective strategy against CP-induced neurotoxicity and cognitive impairment.

## Data Availability

The raw data supporting the conclusions of this article will be made available by the authors, without undue reservation.
